# RaPID: ultra-fast, powerful, and accurate detection of segments identical by descent (IBD) in biobank-scale cohorts

**DOI:** 10.1186/s13059-019-1754-8

**Published:** 2019-07-25

**Authors:** Ardalan Naseri, Xiaoming Liu, Kecong Tang, Shaojie Zhang, Degui Zhi

**Affiliations:** 1 Department of Computer Science, University of Central Florida, Orlando, FL 32816 USA; 2 USF Genomics, College of Public Health, University of South Florida, Tampa, FL 33612 USA; 3 School of Biomedical Informatics, The University of Texas Health Science Center at Houston, Houston, TX 77030 USA; 4 Department of Epidemiology, Human Genetics & Environmental Sciences, The University of Texas Health Science Center at Houston, Houston, TX 77030 USA

**Keywords:** Identity by descent, IBD, PBWT, Random projection, UK Biobank

## Abstract

While genetic relatedness, usually manifested as segments identical by descent (IBD), is ubiquitous in modern large biobanks, current IBD detection methods are not efficient at such a scale. Here, we describe an efficient method, RaPID, for detecting IBD segments in a panel with phased haplotypes. RaPID achieves a time and space complexity linear to the input size and the number of reported IBDs. With simulation, we showed that RaPID is orders of magnitude faster than existing method while offering competitive power and accuracy. In UK Biobank, RaPID identified 3,335,807 IBDs with a lenght ≥ 10 cM among 223,507 male X chromosomes in 11 min.

**Electronic supplementary material:**

The online version of this article (10.1186/s13059-019-1754-8) contains supplementary material, which is available to authorized users.

## Introduction

In the era of precision medicine, very large genotyped cohorts (VLGCs) with rich phenotype information are becoming available. Unlike traditional cohorts, these cohorts contain up to 0.1–1% of an entire large modern population. At this scale, genetic relatedness among samples is ubiquitous. This is well observed in real cohort data [1, 2], recently supported by a theoretical analysis [3] and by a recent high-profile paper on long-range genealogical search [4]. In fact, the genetic relatedness in such datasets may be a rich resource for genetic studies [5].

One piece of direct evidence of genetic relatedness among individuals is identical-by-descent (IBD) segments, i.e., uninterrupted DNA segments inherited from a common ancestor [6]. A unique strength of IBD compared to other population genetics measures is its efficiency for tracking distant relatives. Loss of IBD fragments has an exponential rate as a function of the number of meiosis while decrease in length of IBD fragments is only linear to the reciprocal of the number of meiosis. Even though only a small fraction of distant relatives share IBDs, the expected length of the IBD segments shared between a pair of distant relative can be long, which is easy to validate.

Indeed, identifying relatively large IBD segments (e.g., over 1 cM) among a small number of individuals is considered a “solved problem.” Early methods [7–12] for IBD detection were principally based on statistical models (e.g., hidden Markov models) searching for surprisingly long segments of nearly identical sequences unlikely to arise by chance. While relatively accurate, most methods are slow due to the burden of performing all pairwise comparisons. GERMLINE [9] adopted a computer science approach: it first builds a hash table for all short exact matches (seeds) and then extends them into potential long segments. As a result, GERMLINE has been the fastest IBD detection method since 2009.

By applying these tools to cohorts with dense genotypes, tremendous understanding of genetic relatedness among individuals has been gained. IBD has been used to map the recent genetic ancestry across Europe [13], South America [14], and South Asia [15]. Indeed, large consumer genetic ancestry companies, who own early collections of private VLGCs, were the first to directly study distant relatedness at massive scales. In 2012, for example, ubiquitous distant relatives were detected via IBD segments in both isolated ethnolinguistic populations and in large cosmopolitan “unrelated” populations in about 22,757 customers of 23andMe [2].

However, scaling up IBD detection to very large cohorts is a challenge. For example, in 2017, over 500 million IBD connections among 770,000 customers of AncestryDNA have been reported [1]. In order to generate these IBD calls, AncestryDNA scientists have marshalled tremendous resources and engineering efforts in rewriting the GERMLINE source code, employing large computer clusters and databases, and limiting the method to only detect IBD segments ≥ 5 cM. Therefore, the lack of fast, powerful, and accurate IBD detection methods is the main hurdle between the abundant genotype information in VLGCs and the rich set of IBD-based downstream applications.

Here, we present a new method for IBD segment detection, Random Projection for IBD Detection, RaPID. RaPID is fast because it is based on the positional Burrows-Wheeler transform (PBWT) [16], an extremely efficient index for population genetic sequences, that offers truly linear time search for shared segments in an arbitrarily large cohort. However, PBWT cannot be directly applied to IBD segment detection because it only allows exact matches and does not tolerate single variant mismatches [16]. Real IBD segments are long but require approximate sequence matches due to genotyping errors and mutations.

**Fig. 1:**
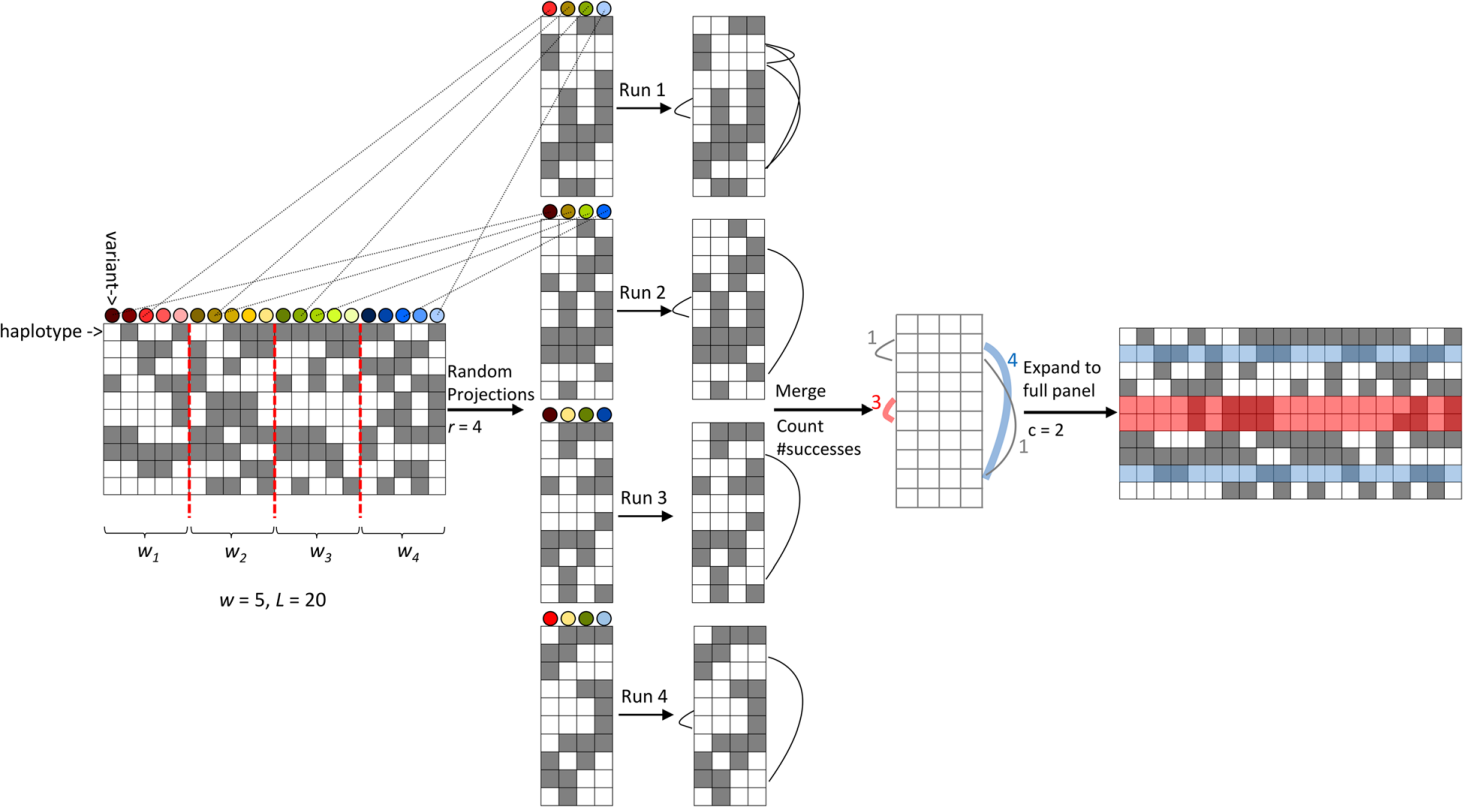
Schematic of the RaPID algorithm. The first step of RaPID is multiple random projections of the input phased genotype panel by selecting a random variant site per window (*w*_*i*_). The second step is running PBWT for each of the projected panels to identify exact matches of subsequences with a length above a certain cutoff. In the third step, exact matches are collected and only those regions reporting more than a certain number are selected to be a candidate IBD segment. The length cutoff (*L*) was set to 10, the window size (*w*) was set to 5, the number of runs (*r*) was set to 4, and the number of successes (*c*) was set to 2. Four different matches were detected, two of them only in one run and two in more than two runs (depicted in blue and red). Two matches with only one success are discarded

The key idea of RaPID is that the problem of approximate high-resolution matching over a long range can be mapped to the problem of exact matching of low-resolution subsampled sequences with high probability. Specifically, given a panel of phased haplotypes as an input, RaPID first produces multiple low-resolution random projections of the original sequences, runs PBWTs finding exact matches over each projection, and then combines the results (Fig. 1). One main advantage of RaPID over the existing methods is that we have a principled way of determining parameter configurations which allows for proper control of detection power and accuracy for the detection of IBDs over a certain target length, given particular marker density and error rates.

Using simulation, we verified that RaPID is much faster than existing methods for typical sequencing and array genotyping data. RaPID also achieves competitive detection power and accuracy for relatively large segments (e.g., ≥ 5 cM) compared to existing methods. Moreover, we evaluated the tolerance of misspecification of parameters of RaPID. Finally, we applied RaPID to the UK Biobank data and identified 3,335,807 IBDs with a length of ≥ 10 cM among the haploid X chromosomes of all 223,507 male participants using 18,857 markers within 11 min, and 8,937,914 IBDs with a length of ≥ 10 cM among the 22 chromosomes of all UK Biobank participants comprising 974,818 haplotypes using 10,911 markers within 29 min, all on a single computing core. For 5 cM runs, RaPID identified about ten times more IBD segments, using roughly proportional more running time.

## Results

RaPID leverages PBWT for speed and random projection for the tolerance of mismatches and control of power and accuracy. PBWT [16] very efficiently finds all exact matches of subsequences at the same locations in different haplotypes with a length greater than *L*, where *L* is the length of the match in terms of the number of variant sites. Given a panel of *M* sequences with *N* variant sites, it can compute all matches with the minimum length of *L* in time *O*(*MN* + *C*), where *C* is the total number of matches. Note that this is optimal in terms of time complexity as *O*(*MN*) is the time for reading the input, and *O*(*C*) is the time for writing the output.

The random projection with PBWT is a general scheme of translating the high-resolution sequences into multiple low-resolution ones, and the approximate high-resolution match can be translated into high probability low-resolution exact matches. The choice of projection function follows the rationale that it should translate similar sequences to the same sequence with high probability while translating dissimilar sequences to the same sequence with a low probability. Separation of the two probabilities can be guaranteed by multiple runs of random projection and PBWT.

In RaPID, we chose random projection as simply picking a random site from a window of fixed size. Parameters of RaPID include (*r*, *c*, *w*), where *r* denotes the number of runs, *c* (≤*r*) the minimum number of runs that a match should be found in order to be considered, and *w* the number of SNPs per window. Given an input panel, we can determine the sequencing error rate *ϵ* and the chances of collision of projections from unrelated sequences *ρ* given *w*. For any target IBD segment length *l* in terms of the number of SNPs, we used probability calculations to choose these parameters to ensure high detection power and low rate of false positives (Additional file 1: Figure S1).

### Benchmarking using simulated haplotypes

To benchmark the detection power, accuracy, and efficiency of RaPID against existing methods, we followed the protocol as in Browning and Browning [11]. Briefly, we generated 4000 haplotypes with a length of 10 Mbps using the macs simulator [17], assuming a population with a history similar to that of the current European population with a mutation rate of 1.3 × 10^−8^ per base pair per generation and a constant recombination rate of 1 cM per 1 Mbps, which results in a heterozygosity rate similar to that of the UK population data. We introduced the genotyping errors in the generated haplotypes at a rate of 0.0025 per genotype for each haplotype [11]. The true IBDs were determined as uninterrupted sharing of the most recent common ancestor above a certain length.

First, we validated that our parameter calculation (as detailed in the “Methods” section) by showing that both high power and high accuracy of RaPID can be achieved with ten runs (*r* = 10) and two successes (*c* = 2) using simulated data (Additional file 1: Figure S2). Using these choices of *r* and *c*, we conducted benchmark experiments to compare RaPID with existing methods.

**Fig. 2:**
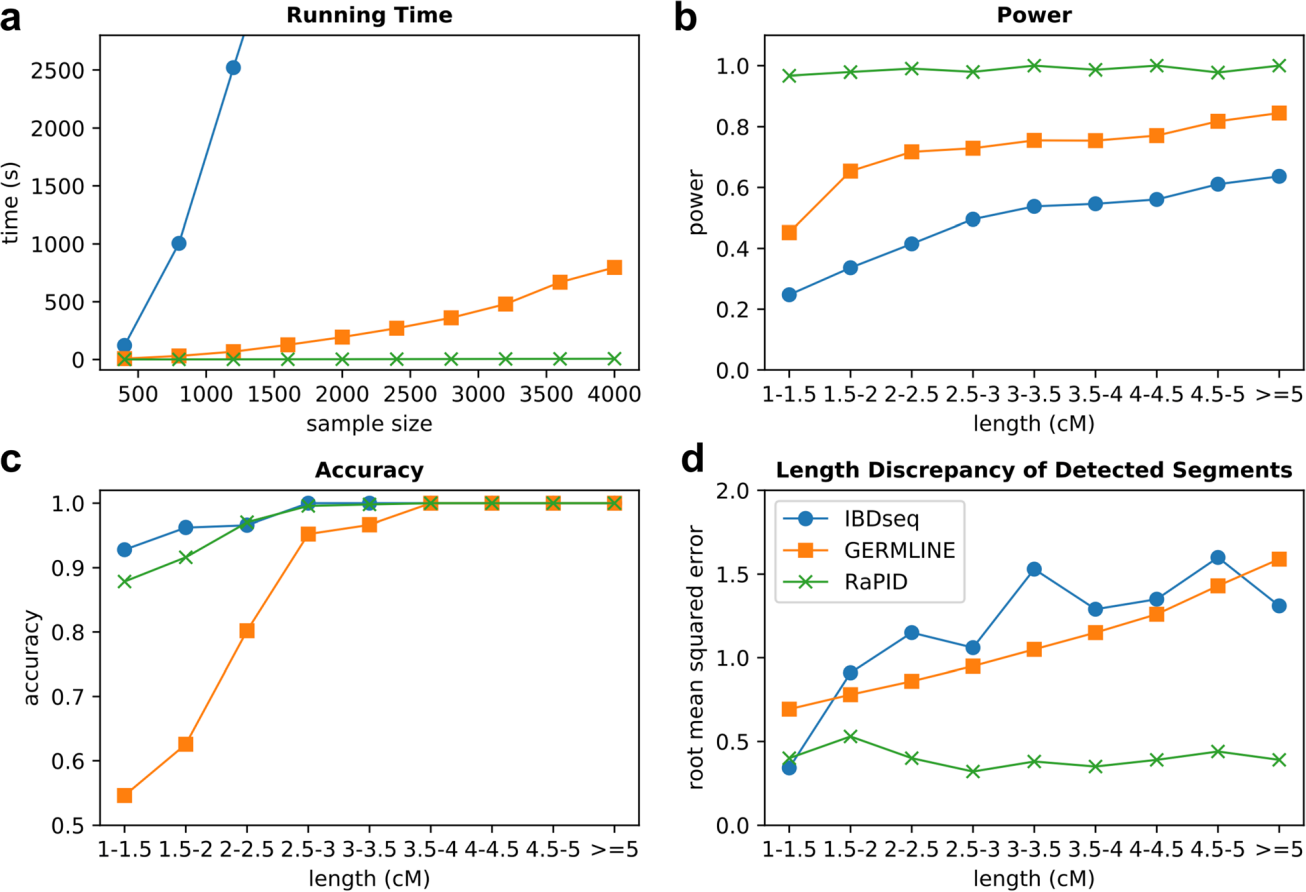
Benchmarking results of RaPID, GERMLINE, and IBDseq for IBD detection in a sequencing platform. Simulated sequences of 4000 haplotypes with a chromosome length of 10 Mbps were generated by macs. **a** Run time: the time for RaPID was for the target length of 3 cM. **b** Power: the average proportion of true IBD segments that have been reported. **c** Accuracy: the number of correctly detected IBDs that share at least 50% overlap with the true IBDs over the number of reported IBDs. **d** Length discrepancy of detected segments: the root mean squared difference in terms of lengths between the correctly detected IBDs and the true IBDs. Parameters of GERMLINE and IBDseq were tuned with due diligence and according to previous literature (see the “Benchmarking using simulated data” section) but may not be fully optimized

Figure 2a shows the run time of different methods for 4000 haplotypes with a chromosome length of 10 Mbps. In our experiments, across IBDs of all lengths, RaPID was much faster than GERMLINE, both of which are much faster than IBDseq. Of note, each of the benchmark runs of RaPID was completed in less than 2 s. RaPID was more than 100 times faster than GERMLINE for detecting IBDs with a target length of 3 cM. For detecting IBDs with a target length of 1.5 cM, RaPID was 25 times faster.

The contrast of run time difference of RaPID and existing methods was even greater for larger samples. IBD detection tools that require pairwise comparison of all haplotypes are generally not scalable for a large number of haplotypes. As a result, they are very slow compared to GERMLINE and RaPID. While the run time for GERMLINE is linearly related to the sample size when the sequences are random, real genetic sequences are related and contain extensive shared segments. As a result, GERMLINE slows down with increased copies of the repeated sequences due to the burden of over-seeding, while RaPID embraces the patterns of sharing thanks to the efficient indexing of PBWT, and maintains a linear behavior (Additional file 1: Figure S3), see the “Computational complexity” section for a detailed time analysis.

Our randomization strategy allows for precise control of desired performance. Assuming the genotyping errors are randomly distributed and given the density of marker, we provide a strategy to select the parameters to target IBD segments of a given length (Methods: Determination of parameters). As shown in Figures 2b and 2c, RaPID exhibited a consistently high power in detecting IBD segments ranging from 1 to 5 cM compared to the existing methods while maintaining a comparable high accuracy. Detecting short IBDs (1–3 cM) is notoriously difficult because at shorter lengths, the number of identical-by-state (IBS) segments among unrelated individuals may overwhelm the signal from the real IBD segments. The accuracy here is defined as the percentage of reported segments where 50% of their length is covered by a true IBD segment, following Browning and Browning [11]. As shown in Additional file 1: Figure S4, the accuracy of RaPID still remains comparable to other tools with a stricter cutoff (75%) and a more relaxed cutoff (25%). It is reassuring to see RaPID achieves high power which is consistent with our theoretical calculation. We ascribed the imperfect level of accuracy to the fact that genotype information across windows was not independent.

An important innovation in the RaPID approach is that RaPID offers a principled way for parameter tuning for different sets. In a simulation study, we investigated the tolerance of misspecification of window size and target length parameters (Additional file 1: Figure S5). We validated that for detecting 5 cM segments, RaPID with both window size and target length optimized for detecting 1.5 cM would severely lose power (0.2). On the other hand, for detecting 1.5 cM segments, RaPID with both window size and target length optimized for detecting 5 cM suffers low accuracy (0.5). Therefore, to maximize power and accuracy for any particular length range, RaPID with accurately determined parameters should be used. However, this indicates that to maximize power and accuracy across all length ranges, RaPID has to be run multiple times using parameters for increasing target segment lengths, and some post hoc process will be needed to assemble segments identified by different runs. If one is willing to sacrifice minor power and accuracy, one can run RaPID with both window size and target length parameters optimized at detecting shorter segments: when we run RaPID with window size optimized for detecting 1.5 cM (*w* = 80), and the target length is fixed *l* = 1.5, we found that the single run offers a reasonable detection power and accuracy for IBD segments across the entire length range of 1.5–5 cM, with very little loss of detection power and about 5% loss of accuracy.

The accurate inference of the length of an IBD segment is often of great interest for population genetics modeling. RaPID also showed smaller length bias compared to IBDseq and GERMLINE (Fig. 2d). Notably, RaPID tended to overestimate the length by about 0.4 cM across all IBD lengths. This is understandable as PBWT reports any streak of windows with matching projections, and it is likely that windows for a true IBD segment be surrounded by several windows with matching projections by chance.

**Fig. 3:**
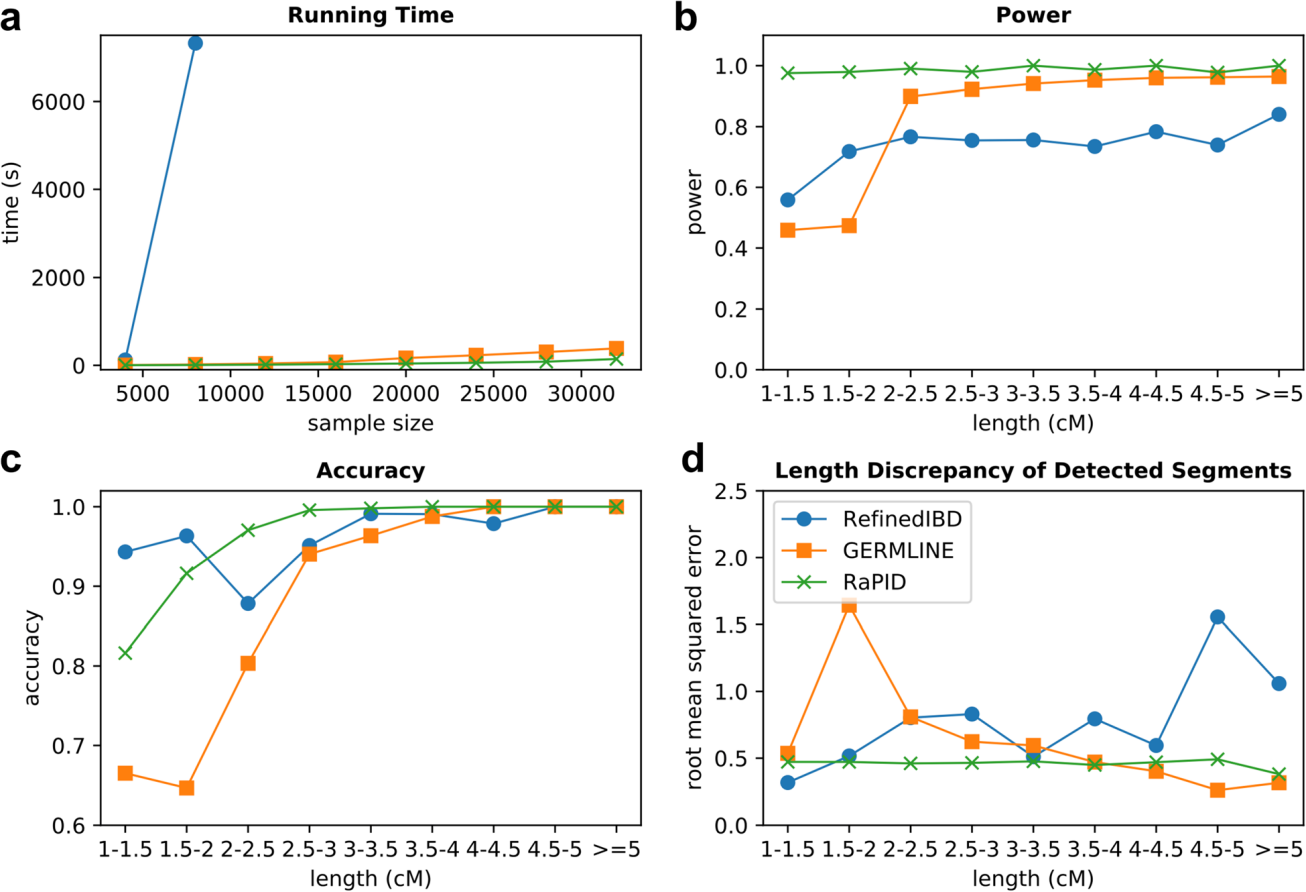
Benchmarking results of RaPID, GERMLINE, and RefinedIBD in a genotyping platform. The simulated sequences with a chromosome length of 10 Mbps were thinned by choosing the variant site with the highest minor allele frequency in every 80 variant sites. **a** Run time for RaPID, GERMLINE, and RefinedIBD with the increasing number of haplotypes from 5000 to 30,000. The target length for RaPID was set to 3 cM. **b** Power. **c** Accuracy. **d** Length discrepancy of RaPID, GERMLINE, and RefinedIBD using 4000 haplotypes. Accuracy, power, and length discrepancy are defined in the caption of Fig. 2. Parameters of GERMLINE and RefinedIBD were tuned with due diligence and according to previous literature (see the “Benchmarking using simulated data” section) but may not be fully optimized

Performance of RaPID in sequencing data can be transferred to other genotype platforms with different marker density and error rates. To demonstrate this, we generated thinned genotyping data resembling that of a typical genotyping platform by choosing the variant site with the highest minor allele frequency in every 80 consecutive variant sites from our simulated data. As shown in Fig. 3b, RaPID has consistently superior detection power over GERMLINE and RefinedIBD. The length discrepancy of RaPID across different target lengths remains constant in the thinned data (Fig. 3d). RefinedIBD has higher accuracy for shorter IBD segments (Fig. 3c) given its haplotype model, but it is significantly slower than RaPID (Fig. 3a). As shown in Additional file 1: Figure S6, the accuracy of RaPID again remains comparable to other tools with a stricter overlap cutoff (75%) and more relaxed cutoff (25%). The accuracy of RefinedIBD with the stricter overlap cutoff remains higher, especially for shorter IBDs. It should be noted that RefinedIBD cannot tolerate genotyping error well. As a result, long IBD segments may be split into multiple short IBD segments. We merged the short IBD segments by allowing a 0.6 cM gap between every two IBD segments and at most two homozygous discordant genotypes, according to the recommendations from the author of RefinedIBD.

Note that RefinedIBD can be applied to sequencing data—but requiring a thinning of genotypes upfront. Since there is no guidance on how best to thin the sequencing data before running RefinedIBD, we decided to include RefinedIBD only in the benchmarking of SNP array data. Nonetheless, since our SNP array data were indeed generated by thinning the simulated sequencing data, we assume RefinedIBD applied to the sequencing data would generate similar results.

Next, we performed several sensitivity analyses of RaPID with more realistically simulated data. A practical concern for real data is the imperfection of phasing. RaPID currently takes phased haplotype data as an input, and thus, its performance may be hurt by phasing errors. To evaluate the tolerance of RaPID for phasing errors, we simulated a population of 200 haplotypes using macs simulator with a chromosome length of 60 Mbps (60 cM) and added two switch errors for each individual. It corresponds to one error in every 20 Mbps, based on an estimate of long-range switch error rate (short 1–3 SNP blips can be considered as mismatches and be handled by the current RaPID framework) in the UK Biobank data from the author of the Eagle software (Table 1 in [18] and personal communication, Po-Ru Loh). As shown in Table 1, the accuracy of RaPID does not change, as expected. The detection power of RaPID also does not change for short IBD segments (1.5–2 cM) while some long IBD segments may be missed (< 50% overlap) which results in a moderate lower detection power for long IBD segments (5–10 cM). However, these long segments are broken by phasing errors and possibly be recovered by post hoc stitching. Therefore, RaPID is robust to long-range phasing errors that are well beyond the detection target length.

**Table 1:** Effect of phasing error on detection power and accuracy of RaPID

Phasing error	Segment length (cM)	Power	Accuracy
None	1.5–2	0.97	0.97
None	5–10	1	1
2 switch error	1.5–2	0.97	0.97
2 switch error	5–10	0.81	1

Another practical concern is how RaPID will tolerate misspecification of the genetic map. While we assumed a constant rate of genetic distances in terms of physical distances in our simulation, the misspecification of genetic distances and also the discrepancy of genetic and physical length are a concern in real data analysis. RaPID can handle variable genetic distances by considering both physical and genetic distances at the same time. Basically, RaPID takes a conservative short target length in terms of physical distance to generate a set of preliminary calls and apply a post hoc filtering by using the genetic map to keep on IBD segments that are above the target genetic distance.

To show the robustness of RaPID with realistic genetic distances, we simulated a population of 4k haplotypes with the genetic distances extracted from the first 10 Mbps of chr1 using the genetic mapping extracted from HapMap Phase II. If we assume constant genetic map and use 5 Mbps as the target length, the accuracy for target lengths larger than 5 cM was 100%, but due to the fast-growing genetic lengths in some regions, the detection power for finding 5 cM segments was only 33% when the minimum target length was set to 5 cM (assuming 1 cM ≈ 1 Mbps). To find a reasonable conservative physical distance target length, we scan the genome-wide genetic map and identify the physical distance such that 90% of the 5 cM segments will be longer in terms of genomic distance. We reached to the target physical length as 1.6 Mbps. When we run RaPID with the target length of 1.6 Mbps and post-processing filter of 5 cM, the detection power increased to 100% while maintaining 100% accuracy. Therefore, we used the 90 percentile physical target lengths for our analyses of UK Biobank data (Additional file 2: Table S2).

We remark that parameter *ρ* is also robust to misspecification. As shown in Additional file 1: Figure S1a, if *ρ* value increases, the detection power does not change but the number of false positives increases if the value approaches one when the other parameters remain the same (e.g., same window size, number of runs, and number of successes). Therefore, RaPID is robust to misspecification of *ρ* to a lower value. In data analysis, we choose to use the 90 percentile of *ρ* in all windows to provide a safety net for misspecification.

### Revealing dense genetic relationships among UK Biobank participants

To demonstrate the run time and quality of IBD segment calls of RaPID, we applied RaPID to the genotypes of the UK Biobank participants. We expected to identify extensive genetic relationships in this very large cohort. In this study, we chose the X chromosomes of all 223,507 male participants to benchmark RaPID without the potential complications due to phasing inaccuracy. We also run RaPID for chromosome 22 of all phased haplotypes of 487,409 participants. Based on our estimate, the low phasing error rate of UK Biobank haplotypes should only have a minor reduction of power. We limited the detection of IBD segments with lengths ≥ 5 cM and ≥ 10 cM. Notably, ≥ 5 cM is the range most informative for genetic genealogy. In expectation of complexity in real data, we used conservative parameter settings: (i) physical distance cutoff to cover 90% of target genetic length and (ii) estimated *ρ* that is greater than 90% of windows. While these settings will reduce the running speed, we expect that the power and accuracy should be quite high even with imperfect phasing (one major switch error per 20 Mbps). The running parameters were documented in Additional file 2: Tables S1 and S2. A tally of descriptive of these runs is shown in Additional file 2: Table S3. We will focus on the results of the ≥ 5 cM runs, while the results of ≥ 10 cM runs are mostly available in Additional file 1: Figures S7-S8.

**Fig. 4:**
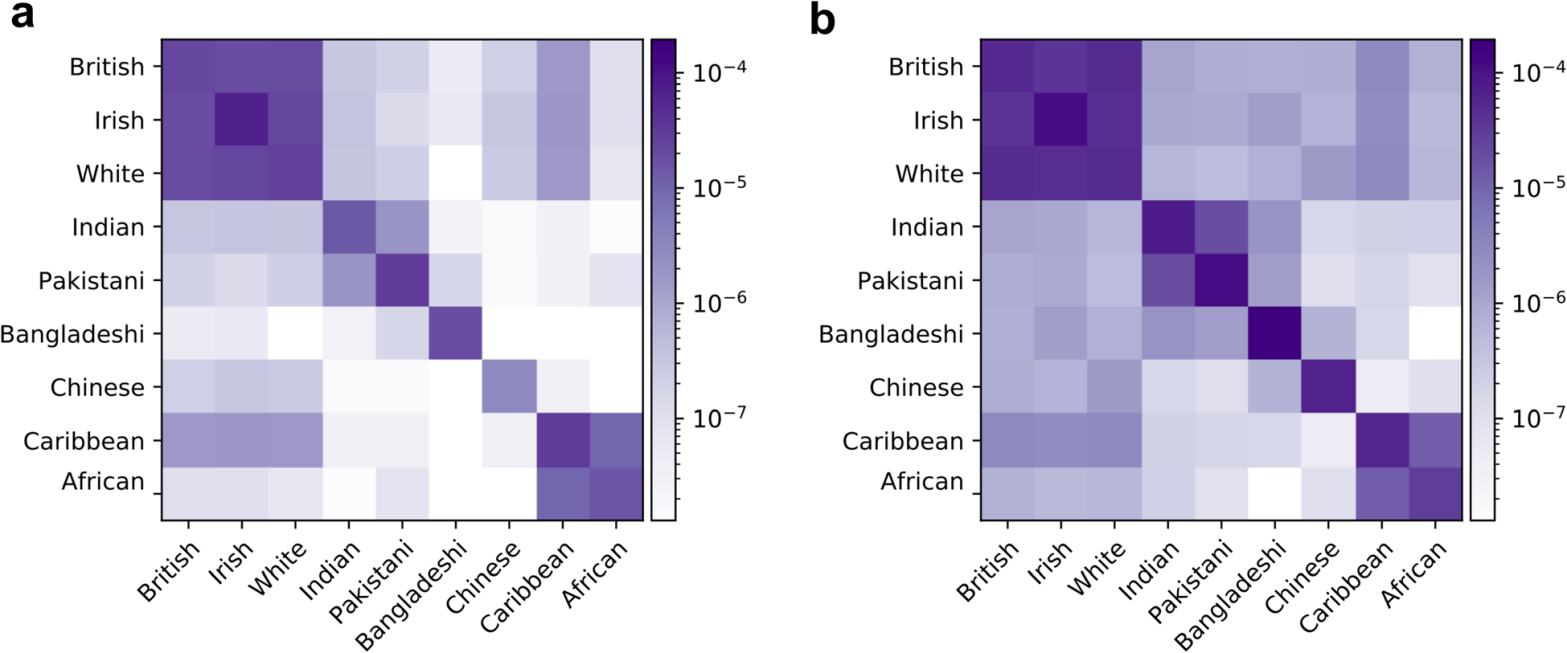
Kinship values (in log scale) among different ethnic groups in the UK Biobank using detected IBD segments with a length of 5 cM and above in chromosome 22 (**a**) and chromosome X (**b**). The ethnicity groups were extracted using self-reported data by the UK Biobank participants

First, we plotted the average kinship based on the identified IBD segments among nine self-reported ethnic groups excluding individuals who reported multiple racial/ethnic groups (Fig. 4). The objective was to verify whether more IBD segments are detected within each group versus between different groups. As expected, IBDs within ethnic groups were stronger than that between ethnic groups. Also, IBDs within each racial group were stronger than that between racial groups. Moreover, the patterns were grossly consistent between chromosome 22 and chromosome X. Nonetheless, chromosome X showed stronger IBD signals, reflecting that chromosome X typically undergoes less meiotic recombinations than autosomes.

Further, we compared the IBD segments identified by RaPID to the genetic relationships. Notably, up to third-degree relatedness, including MZ twins, parent-offspring, full sibs, second degree (half sibs and avuncular), and third degree (e.g., first cousins) were extracted from UK Biobank-distributed files [19] which was originally generated by UK Biobank team using the KING program [20] applied to genotypes over a filtered set of markers across the genome. Note that KING estimated the global average IBD at SNP level and did not estimate any IBD segments.

**Fig. 5:**
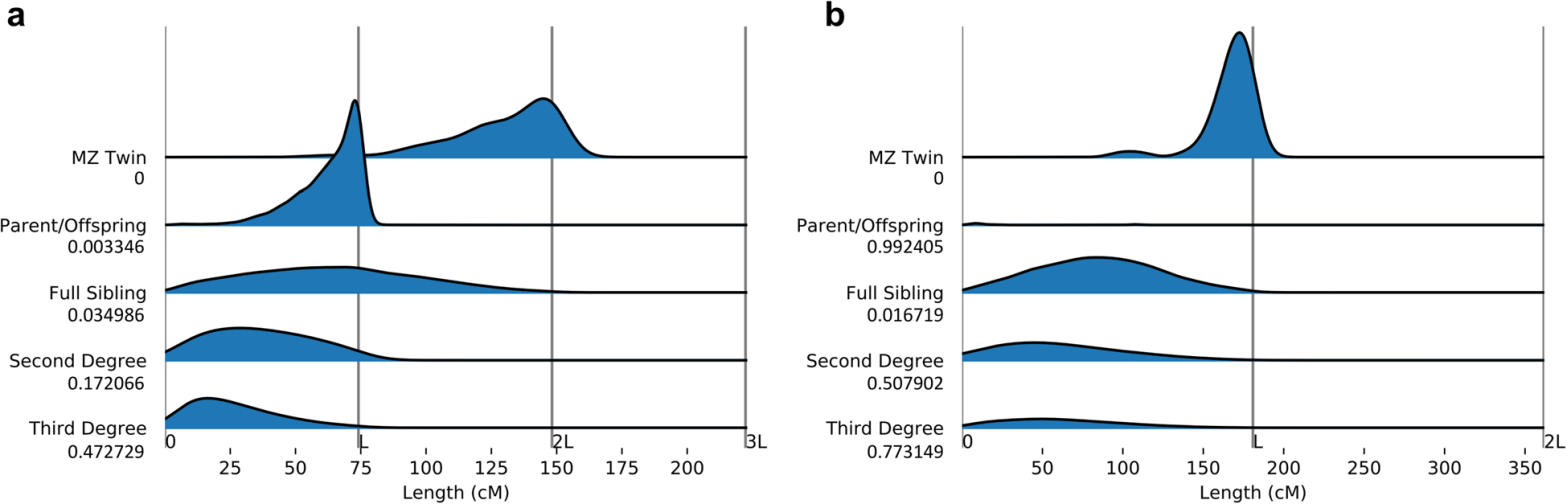
The probability distributions of the sum of genetic lengths shared among pairs of individuals in five types of relatedness using detected IBD segments by RaPID with a target length of 5 cM and above in chromosome 22 for all participants (**a**) and chromosome X for male participants (**b**). The numbers below each category show the proportion of pairs for which no IBD segment over 5 cM were detected by RaPID. *L* denotes the total length of the chromosome in cM. Plots were generated by joyplot python package with Gaussian kernels smoothing (default gaussian_kde of scipy)

For all pairs of individuals within third-degree relatedness, we plotted the distribution of the total length of IBD segments among each pair stratified by their relationships (Fig. 5). We observed that the length distributions were largely consistent with expectations. Notably, for a significant proportion of pairs, no IBDs were observed. We validated these proportions through a simulation study following the type A simulation of [21].

**Fig. 6:**
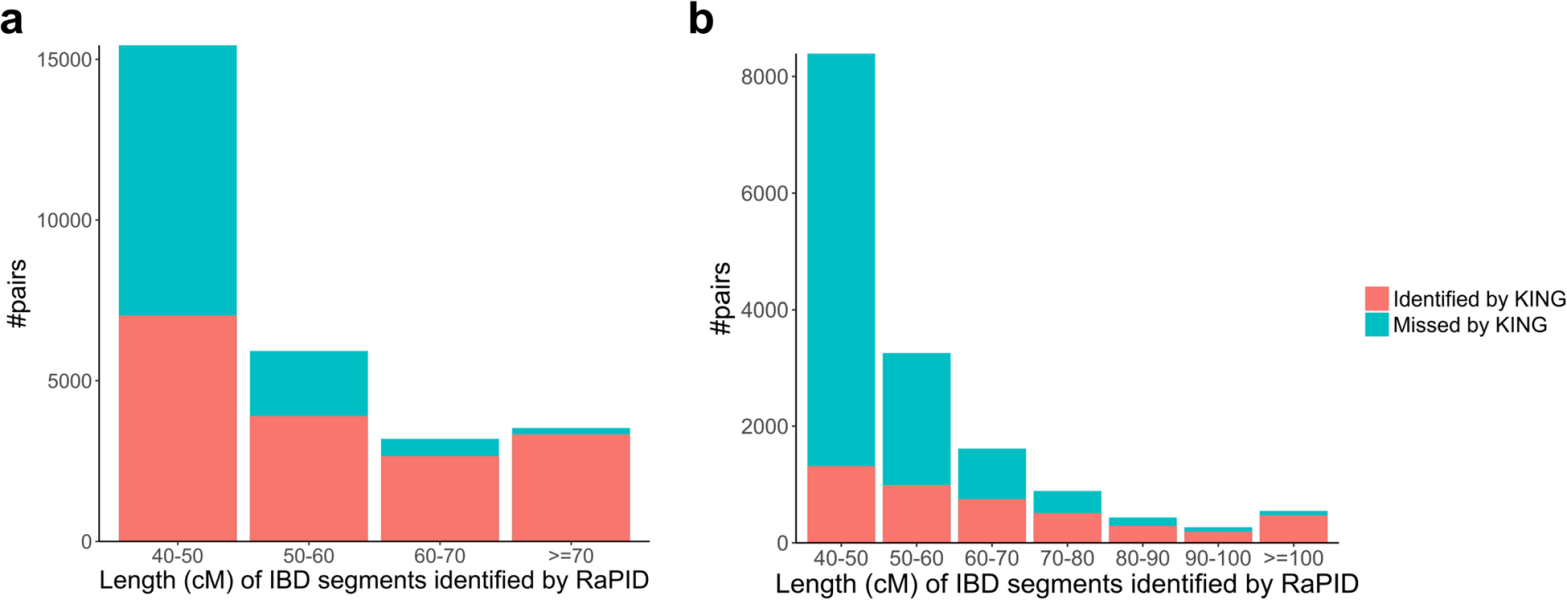
The number of individual pairs with IBD segments identified by RaPID in chromosome 22 (**a**) and chromosome X (**b**), stratified by IBD length, and the number of such pairs that are also identified by KING

Interestingly, RaPID identified a large number of pairs of individuals who share an IBD segment over 40 cM yet not included in the UK Biobank KING results. As shown in Fig. 6b, while KING identified 86% of the pairs in chromosome X with IBD length ≥ 100 cM identified by RaPID, KING missed 79 pairs with IBD segments ≥ 100 cM. We verified that each of these segments demonstrated a much lower mismatch rate within the segments than outside of the segments (Additional file 2: Table S4). This paradox is however possible because two individuals separated by more than three meiosis over the entire 196 cM X chromosome have a small but non-zero probability to have zero crossovers over the total 588 cM. Moreover, only 16% of the pairs with IBD length 40–50 cM in chromosome X were identified by RaPID while 46% of pairs with IBD length 40–50 cM in chromosome 22 had been reported by KING. This is again plausible because most observed IBD segments with a length of 40–50 cM may be from distantly related pairs of individuals that are separated by > 3 meiosis. Therefore, our results highlighted the expected discrepancies between the genealogical relationships and the realized sharing of IBD segments. Of note, chromosome 22 results between RaPID and KING are more consistent—only about 54% of the pairs sharing a 40–50 cM segments are not detected by KING. The main reason is probably that for the same length, IBDs in chromosome X indicate deeper genetic relationships than that in autosomes. In general, our results showed that IBD-based methods for genetic relationship inference would reveal more genetic relationships in biobank-scale datasets.

## Discussion

While the high efficiency of RaPID stems from the PBWT data structure [16] which offers computational complexity linear to the sample size and the output size, our main contribution is the randomization scheme that translates the problem of identifying high-resolution approximate matches to the problem of identifying low-resolution exact matches with high probability. As a result, RaPID not only tolerates mutations and genotyping errors, but also controls power and accuracy more effectively than existing methods. Through benchmarking experiments, we also evaluated the robustness of RaPID to misspecifications of parameters. We showed that with parameters determined by a data-driven fashion, RaPID can achieve high power and accuracy even with uneven genetic distance, variable linkage disequilibrium, and marker density.

One of the caveats is that RaPID is not designed to identify very short IBDs. For very short IBD segments, a very high number of runs (*r*) will be needed to achieve high accuracy, and thus, RaPID is no longer efficient. Compared to the current implementation of RaPID, traditional methods are probably more accurate for shorter IBD segments.

One of the biases of RaPID is that it tends to overestimate the length of IBD segments. The overestimation bias is largely a result of the nature of our algorithm. While a true IBD segment leaves a core footprint of a continuous streak of matching windows, because of the random chance of window matching (*ρ*), the core footprint on average will be over-extended by 1/(1 − *ρ*) windows. This bias could be corrected by trimming at the ends of a IBD segment to maximize some objective functions, though not without incurring extra computational cost. We will work on this issue in future development.

Our result using RaPID offers a peek into the massive genetic relationship in the UK Biobank. For example, we identified about 3 million IBD segments with lengths over 10 cM in 223,507 X chromosome haplotypes in the UK Biobank, revealing massive genealogical information in biobank cohorts. Our tool opens the possibility to study the genealogical details of such populations.

We demonstrated that applying RaPID to biobank data is practically feasible in terms of resources. Our experiment identifying IBDs over 10 cM for all male X chromosomes only took 11 min on a single core of a desktop computer with 0.9-GB peak memory usage. We extrapolate the run time for identifying IBDs with length ≥ 10 cM for the entire genome for all participants of the UK Biobank would be about 12 h. The run time for identifying shorter IBD segments will be longer, mainly due to the fact that there are more segments to be identified and reported. Although we believe that our efficient method can generate IBD segment calls in very large samples, and this will be a useful resource for the community, this endeavor will require substantial additional research and resources on rigorous evaluation of the IBD calls, which is beyond the scope of current work.

With the availability of very large genotyped cohorts, it is the first time in human history that we can directly observe the dense genetic relationships in large populations via individual IBD segments. With RaPID making such information explicit, new research avenues may be enabled. For example, more powerful genetic association studies may be possible with explicit representation of shared haplotypes among individuals and with more direct representation of genetic relatedness among millions of individuals. Further, IBD information in large cohorts offers unprecedented details about population demographic in the past 1–50 generations [1]. In addition, RaPID can be used to identify runs of homozygosity (ROH) tracts [22]. We expect RaPID and biobanks will open new avenues of human genetics research such as IBD mapping [5].

## Conclusions

In this paper, we present a fast and accurate method for detecting IBD segments in cohorts with dense genotype data. Our method, RaPID, can detect IBD segments across a wide-length spectrum very efficiently while achieving superior accuracy and power compared to existing methods. Moreover, with a principled way of parameter tuning, RaPID is adaptable to data from different genotyping platforms with varying marker density and error rates. We showed that RaPID provides a clear advantage over existing methods on detecting relatively large segments (≥ 5 cM) for large biobank-scale data.

## Methods

### Brief description of PBWT

PBWT [16] provides a fast method for finding all matches with a length greater than *S*, where *S* is the length of the match in terms of the number of variant sites. Given a panel of *M* sequences with *N* variant sites, it can compute all matches with the minimum length of *S* in *O*(max(*MN*, number of matches)) time. The PBWT algorithm can also find all maximal matches in *O*(*MN*). A sequence *s* has a maximal match in [*k*_1_, *k*_2_] to *x*_*i*_ (*i*th sequence in the panel) if the match *x*_*i*_[*k*_1_, *k*_2_] =  = *s*[*k*_1_, *k*_2_] cannot be extended, and there is no longer match in the panel that includes [*k*_1_, *k*_2_]. The basic idea of PBWT is to sort the sequences by their reversed prefix at each position. The algorithm sweeps through the list of variant sites and keeps the starting positions of the matches between neighboring prefixes. The algorithm can be used to find IBD segments in large cohorts very efficiently in terms of time and space. However, as pointed out by Durbin, PBWT searches for exact matches and it does not account for genotyping or phasing errors. One may use shorter matches as seed and extend the matches, similar to the approach adopted by GERMLINE. However, choosing an appropriate length as seed is not trivial. In particular, short seeds will result in many matches, and the run time will increase dramatically.

### Random projection PBWT tolerates errors and mutations

Our insight for IBD detection comes from the idea that IBD segments are approximate high-resolution matches over a long range, which can be mapped to a problem of exact matches of low-resolution sequences with high probability. Specifically, we designed a randomized algorithm that first produces multiple low-resolution PBWTs on random subsets of markers and then combines the results efficiently using an interval tree data structure. Multiple PBWT runs are needed because a single run of PBWT on randomly selected markers usually will have low power and accuracy.

In order to tolerate mismatches, we divide the original haplotype sequences into windows of equal sizes and use a projection function *y* = *f*(***x***) that translates a long bit-string vector ***x*** **=** (*x*_1_, *x*_2_, …, *x*_*p*_) over a window into a single bit *y*. As a result, each original haplotype sequence is translated into a scaled-down lower-resolution sequence. We built PBWT over the translated sequences to identify matches efficiently.

### Desired properties of a projection function

The projection function *f* should satisfy the following properties so that the algorithm can function well:

1. *f* should be fast to compute.
2. *p*_+_, the probability that *f* translates similar sequences to a same bit, is high (*p*_+_ ≈ 1), i.e., error tolerance.
3. *p*_−_, the probability that *f* translates dissimilar sequences to a same bit, is low, and *p*_−_ < *p*_+_.
4. *f* in each repeated round should be independent or at least carry different information.

Because of (2, 3, 4), we can run a total of *r* rounds of random projection. Consider that true IBD spans *L* windows, and *f* in each round is independent, the number of times each of *L* windows hits a same bit follows ${X}_T\sim \mathrm{binomial}\left(\mathrm{r},{p}_{+}^L\right).$ Similarly, the *L* window match can happen to a random pair of sequences with ${X}_F\sim \mathrm{binomial}\left(\mathrm{r},{p}_{-}^L\right).$ Note that *p*_−_ does not need to be very small as long as there is a gap between *p*_+_ and *p*_−_. With increasing *r*, *Pr*(*X*_*T*_ > *X*_*F*_) → 1, we can choose a constant *c* such that *Pr*(*X*_*T*_ > *c*) → 1 and *Pr*(*c* > *X*_*F*_) → 1. Hyperparameters, including the window size *w*, the total round number *r*, and the required number of hits *c*, can be tuned by probability calculations, given *p*_+_ and *p*_−_.

### The choice of projection function in RaPID

The projection function *f* computes a representative value for a set of SNPs. Assume the projection function *f* selects a SNP in each window at random, then as a result, *p*_*+*_ *=* 1 *− ε*, where *ε* is the error/mutation rate and *p*_*−*_ *= p*^2^ *+* (1 *− p*)^2^, where *p* is the minor allele frequency. For RaPID, we chose a random SNP in each window weighted by its minor allele frequency. There might be other choices of projection functions. For some other applications, one may want to put more emphasis onto rare variants, SNP density, or base it on haplotype frequency information.

### Parameters of RaPID

Parameters relevant to the binomial models of RaPID are (*r*, *w*, *c*), where *r* denotes the number of runs, *w* the window size, and *c* (≤*r*) is the minimum number of times that a match should be found in order to be considered. We run random projection *r* times, with each window containing *w* SNPs, and then run PBWT and consider any returned match with ≥*S* SNPs as a “hit.” The number of hits follows a binomial distribution for both true IBDs and non-IBDs. For a true IBD segment pair of length *l* with identical original sequence, we assume error rate of *ε*(≪1), including both mutation and genotyping error rate, the binomial probability in each run is ${\left(1-\varepsilon \right)}^{\frac{S}{w}}\approx {e}^{-\frac{S\varepsilon}{w}}$, or *X*_*T*_~binomial(r, *e*^−*Sε*/*w*^). For a random pair of segments, the probability of having a hit with at least *S*/*w*-window is ${\rho}^{\frac{S}{w}}$, where *ρ* = 1 − *p*_ is the probability that a randomly chosen pair of chromosomes would share the projected sequencing in a window, or ${X}_F\sim \mathrm{binomial}\left(\mathrm{r},{\rho}^{\frac{S}{w}}\right)$, where *X*_*T*_ denotes the number of runs that a true IBD segment has been reported. *X*_*F*_ denotes the number of runs that a false IBD segment has been reported. The success of random projection PBWT relies on the choice of a parameter *c*, such that the power Pr(*X*_*T*_ ≥ *c*) is high, while the expected number of false positives $\left(\genfrac{}{}{0pt}{}{N}{2}\right)\Pr \left({X}_F\ge c\right)$ is low. Note that *ρ* is a population genetics parameter determined by haplotype frequency, which varies from region to region. *ε* is determined mainly by genotyping error rate as mutation rates are typically much lower. *ε* is in the range of 0.001–0.01.

### Merging of PBWT runs

In order to prevent false positives, only those hits that occur at least *c* times are considered true hits. However, it is very unlikely that the starting and ending positions of two different hits from different runs are exactly the same. Instead of checking the exact starting and ending positions, we consider the overlap between two hits. The outputs of different runs should also be merged together even if the value *c* is set to one to remove redundant hits from different runs.

Each output of the PBWT run contains the indices of two haplotypes, *k*_1_ and *k*_2_, starting and ending positions of the match. To filter and merge the intervals efficiently, interval trees are used. An interval tree can be constructed in *O*(*t* log *t*) for *t* intervals and queried in *O*(*h* + log *t*), where *h* is the number of overlapping intervals. The large number of exact matches during each run requires an efficient method to merge the results. In order to compare the outputs, we sort the hits from each run by their indices using *Counting sort.* This sorting has a time and space complexity of *O*(*n* + *k*), where *n* is the number of entries and *k* the maximum key value*. Counting sort* is highly time- and space-efficient for sorting hits. Note that the maximum key value is the total number of individuals or haplotypes while the number of hits is usually larger than the total number of haplotypes. We sort the keys first based on their second index *k*_2_ and subsequently based on their first index *k*_1_. As a result, the time complexity of sorting the reported segments by their key pairs will be *O*(*n* + *k*).

**Fig. 7:**
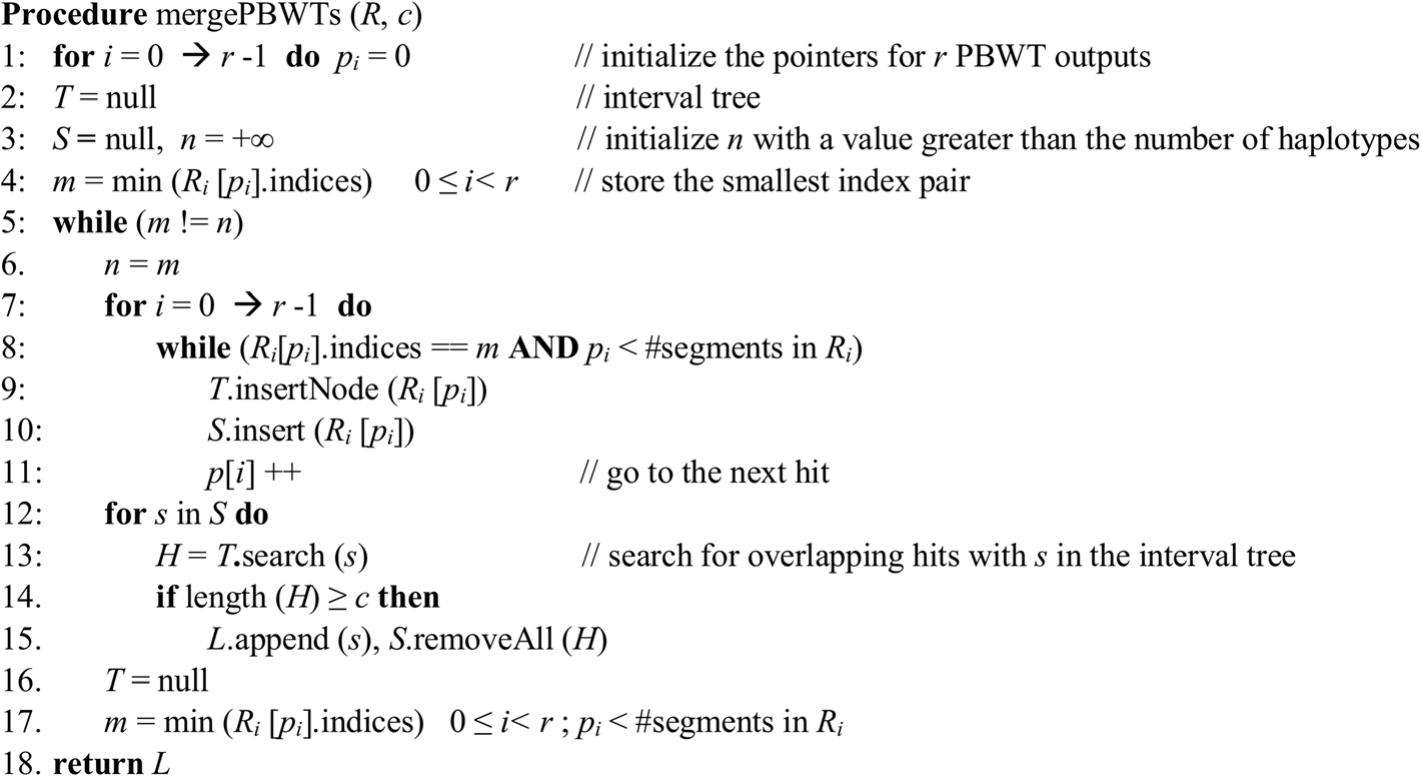
Pseudo-code for merging the results of multiple PBWT outputs. *R* is the set of outputs, *R* = {*R*_0_, *R*_1_, *R*_*2*_, …, *R*_*r*−1_}, and *c* is the minimum number of occurrences

While merging the outputs of multiple runs, memory usage is crucial, since each output may contain millions of entries. We used pointers to extract the hits simultaneously from different runs that are stored in separate files. Assume the outputs are sorted based on *k*_1_ and *k*_2_ and *k*_1_ <*k*_2_. For each PBWT output *i*, a pointer variable *p*_*i*_ is used to point to the current hit that is being processed. A global variable *m* contains the minimum value of (*k*_1_, *k*_2_) pair. *R*_*i*_ denotes the results of the *i*th output of PBWT. *R*_*i*_[*p*_*i*_] is added to the current interval tree and a set *S*, as long as *R*_*i*_[*p*_*i*_] is equal to *m* and *p*_*i*_ is increased by 1. If none of the *p*_*i*_ variables change, then each element in *S* is searched in the interval tree. A hit is stored if the number of overlapped intervals exceeds the given threshold *c*. The remaining hits are then discarded, and the variable *m* is updated. Figure 7 illustrates the pseudo-code for merging the outputs of multiple PBWT runs. The procedure mergePBWTs gets a set of PBWT outputs sorted by their indices and the parameter *c*. It computes the hits that occur at least *c* times out of *r* different PBWT runs.

### Computational complexity

For any algorithm for reading the *M*-by-*N* haplotypes in the panel, and writing *C* detected IBD segments above a certain target length *L*, the minimum time complexity is *O*(*MN* + *C*). RaPID uses PBWT which is *O*(*MN*), and thus, the overall complexity is *O*(*MN* + *C*). As *L* is large enough, *C* shrinks to an extent such that *O*(*MN*) is the larger term, and thus, the overall complexity becomes *O*(*MN*).

This result is in contrast with seed-and-extension methods such as GERMLINE. GERMLINE uses a hash table that bookkeeps all seed matches and is forced to extend all these seed matches even though not resulting in long IBD segments. This would be even exacerbated in a modern population that had undergone an exponential growth in the recent past. On the other hand, PBWT-based RaPID is less affected by these frequent short matches. In addition, the space complexity is proportional to the size of the hash table. Therefore, RaPID has a superior time and space complexity to seed-and-extension methods when the target length is long enough (e.g., ≥ 5 cM).

### Determination of parameters

The parameters, the number of runs *r*, and the minimum number of passes *c* will determine the appropriate range of subsampling values that will result in high true-positive and low-positive probabilities. The value of *r* should be sufficiently large to show a clear separation between true and false positives, at the same time be small enough to curb the run time burden.

We use a numerical optimization to calculate the optimal parameters for an input haplotype panel for a target IBD length. We define an objective function to be minimized using true-positive *t*_*p*_ and false-positive *f*_*p*_ rates:

$$ \mathrm{Given}\ \lambda, M,d,L\ \mathrm{and}\ \varepsilon, \mathrm{find}\ \varTheta =\left\{r,c,w\right\}\ \mathrm{s}.\mathrm{t}.f\left(\varTheta \right)=\lambda {f}_p-{t}_p\ \mathrm{is}\ \mathrm{minimized}, $$

where *t*_*p*_(Θ)~binomial(*r*, *c*, *e*^−*Lε*/*w*^), ${f}_p\left(\Theta \right)\sim \mathrm{binomial}\left(r,c,{\rho}^{\frac{L}{w}}\right)$, and *ρ* = *g*(*w*). *M* denotes the number of haplotypes, *d* the marker density, *L* the minimum length in terms of the number of sites, and *ε* the expected genotyping error rate. *t*_*p*_ and *f*_*p*_ can be calculated using binomial distributions using additional parameters that are to be estimated, namely *r*, *c*, *w*, and *ρ*. *λ* adjusts the weight of false-positive rates. We set $\lambda =\frac{M\left(M-1\right)}{2}$ which is the number of total comparisons. As noted above, *r* denotes the number of runs, *c* the number of successes, and *w* the window size. The parameter *ρ* denotes the random probability of a match. The true-positive rate (or detection power) is independent of the parameter *ρ*. True positive is primarily a function of *d*, *L*, *ε*, *r*, *c*, and *w*. As we increase the number of runs (*r*) and window size (*w*), the true-positive rate should also increase. However, large window sizes or the number of runs (while keeping *c* constant) will result in higher false-positive rates and consequently lower accuracy. False positive depends on the expected random probability of a match (*ρ*) rather than the error rate. Furthermore, *ρ* can vary depending on the selected window size.

Given a dataset, *ρ* and *w* are functionally linked, and their link *ρ* = *g*(*w*) is determined by the input data as follows: *ρ* is basically a function of minor allele frequencies, assuming the number of single matches at random is significantly higher than those within true IBD segments. Simply, we can define *ρ* with the formula:

$$ \rho ={p}^2+{\left(1-p\right)}^2, $$

where *p* denotes the minor allele frequency (MAF) of the SNP that we sampled from the window. Since we use a weighted random projection by giving more weight to the sites with the higher MAF, we can compute the expected MAF of the selected SNP for each window. As a result, the MAF may change by using different window sizes. On the other hand, the changes of expected MAF will be small at a sufficiently large window cutoff in population data (as shown in Additional file 1: Figure S9). To choose a more conservative value for *ρ*, we pick the 10th percentile of expected MAF (90th percentile of *ρ*) along the chromosome.

The objective function is optimized as follows: First, we scan the input panel and determine *M*, *d*, and the functional link *g*. Second, we choose *r* = 10, and *c* = 2, since based on our experience, an optimal solution with *t*_*p*_ ≈ 1 and *f*_*p*_ ≈ 0 can be found with these choices. We set *ε* = 0.0025 as this is the maximum expected error rate and often used by existing methods. Third, we scan all window sizes from 1 up to the maximum window size of 500. This is because we found that, usually a range of parameters can achieve the minimum value for the objective function within numerical precision. We thus report the permissible range. Any window size within this range is permissible. If more than one window size is available in the permissible range, we manually pick a window size by the following practical concerns. As shown in Additional file 1: Figure S1a, if there is a miscalculation of *ρ* and its value is close to 1, then smaller window sizes would not cause higher false-positive rates. Hence, we recommend picking smaller value within the permissible range, especially for panels containing a high number of sites with very rare minor alleles, e.g., sequencing data. Finally, after choosing the window size, we verify that the corresponding *t*_*p*_ ≈ 1 and *f*_*p*_ ≈ 0. If this is not verified, we will go back and pick larger *r* and repeat this process.

Please note that the most time-consuming task is actually to scan the input file and compute the MAFs. If MAFs were already provided, the computation can greatly be sped up. Scanning the window sizes and computing the true and false-positive rates can be accomplished within a few seconds using regular PCs. The minimum window size is the starting window size that will result in high detection power assuming the maximum error rate was estimated correctly or is lower than expected. Note that the true positive is independent of the parameter *ρ*. The maximum reported window should still result in high accuracy, assuming the estimated *ρ* value is not much higher than the estimated one. By default, the program aims to find the points where the objective function is equal to − 1, where *t*_*p*_ and *f*_*p*_ are rounded by two digits.

We provide a script that implements the optimization procedure described above (see the “Availability of data and materials” section). The number of runs and successes can be changed manually if the script cannot find any permissible window for the given data. We also reported the permissible window ranges and the chosen window size of our experiments in Additional file 2: Table S2.

In order to estimate the parameters, the minimum length of IBD segments should be given. Each run of PBWT algorithm will produce a list of the matches that exceed a given length. The length is defined in terms of consecutive variant sites. Assuming the variant sites are distributed evenly in the chromosome, then the average number of variant sites to gain the minimum length can be computed easily by *S* =  ⌊*l* × *d*⌋, where *l* is the minimum IBD length in Mbps, and $d=\frac{T}{chr\_\mathrm{length}}$ where *T* denotes the total number of variant sites in the chromosome, and *chr* _ length denotes the length of the chromosome in Mbps or cM. In order to find IBD segments larger than a given length in cM in real data with various recombination rates, RaPID can handle genetic and genomic distance at the same time. The parameters can be selected using the minimum target length in terms of Mbps. The genomic distance in terms of Mbps can be picked as the minimum physical distance to cover at least 90% of the length in cM. RaPID will search for the matches greater than the given length in terms of Mpbs and ignore those that are shorter than the given length in terms of cM.

### Benchmarking using simulated data

We generated 4000 haplotypes 10 Mbps in length and their ancestry trees using macs simulator [17], assuming a population with a history similar to that of the current Europeans with a mutation rate of 1.3 × 10^−8^ and a constant recombination rate (1 cM per 1 Mbps) [11]. To simulate the genotyping error in the generated haplotypes, we inserted an error rate of 0.0025 for each haplotype as in [11], corresponding to the genotyping quality of 26 in standard VCF files. The true IBDs were determined as [11]: we sampled the ancestry trees at every 5 Kbps, and if the most recent common ancestor of a pair of individuals remains constant, the corresponding segment of the pair was considered as true IBD.

In order to evaluate the correctness of detected IBDs, we computed the accuracy and power of RaPID, GERMLINE, and IBDseq for the simulated data of 2000 individuals. GERMLINE uses a seed-and-extend approach to detect IBD segments, and IBDseq applies a statistical approach that is designed to analyze unphased data. PARENTE2 [23] is designed to handle unphased data which employs a window-based approach, where the windows contain non-consecutive, and randomly selected markers. It aggregates multiple haplotypic models to estimate the likelihood of IBD segments. We were not able to run PARENTE2 on our simulated data of 2000 individuals on a computer with 128 GB memory. We ran PARENTE2 on 10% of our simulated population and it used ~ 10 GB of memory. The running time was also significantly worse than GERMLINE and our method. It took 5480 s for 10% of the simulated data. PARENTE2’s running time and memory use scales linearly with the chromosome length, but scales quadratically with the population length. As a result, we may expect approximately 548,000 s or 152 h to run the program on our entire simulated data.

Accuracy is defined as the percentage of reported IBDs with at least 50% overlap with a true IBD among the total number of reported IBDs. Power is defined as the average proportion of true IBD segments that have been reported. We also computed the length discrepancy of called IBD segments which is directly correlated with the total IBD length between a pair of individuals, and thus directly relevant to our analysis of kinships. Subsequently, we demonstrated the efficiency of RaPID regarding run time with an increasing number of haplotypes.

To benchmark array genotyping as input, the simulated data of 4000 haplotypes with an error rate of 0.0025 were thinned by choosing a window size of 80 variant sites and selecting the variant sites with the highest minor allele frequency in each window. We computed the accuracy and power of RaPID, GERMLINE, and RefinedIBD for the thinned simulated data. The parameters of RaPID were then selected according to the thinned dataset. Additional file 2: Table S1 provides a detailed list of used parameters for the benchmarking.

We tried to apply due diligence to tune the parameters for all other tools during benchmarking. For GERMLINE, we used the tag -h_extend in order to prevent extensive false positives. With -h_extend tag, GERMLINE assumes that the data are well-phased and does not try to extend matches from both haplotype pairs at the same time. Basically, it considers each haplotype independently. By default (without using -h_extend), GERMLINE will usually produce a large number of false-positive matches due to the extension of matches from any of the haplotypes between two pairs. For genotyping data, the default parameters of GERMLINE only gave a small number of IBDs in output. We thus tuned the word size for GERMLINE to get a similar number of IBD segments as in the ground truth to balance the power and accuracy. For RefinedIBD, we followed recommendations from the author: we first searched for shorter IBD segments using a very lenient LOD score cutoff of 0.5 as the program cannot handle genotyping error well and then used an additional script for RefinedIBD to stitch the results. Finally, for IBDseq, we set the error rate parameter to the actual error rates used for generating the simulated sequences. Detailed information about the parameters is included now in Additional file 2: Table S1. On the other hand, RaPID has a principled way of choosing parameters given target segment length, genetic similarity (*ρ*), and mismatch rate, and we did not tune the parameters beyond what guided by the principles.

### Analysis of the UK Biobank data

The genotype data of the UK Biobank (version 2) data [19] were extracted. RaPID was applied to the extracted data to find IBD segments with a minimum length of 5 and 10 cM. The number of runs was set to 10 and the minimum number of passes to 2. The window sizes of 3 and 5 SNPs were selected for 5 and 10 cM, respectively (see Additional file 2: Table S2).

The genetic map generated by the Phase II HapMap (available at ftp://ftp.hapmap.org/hapmap/recombination/2011-01_phaseII_B37/genetic_map_HapMapII_GRCh37.tar.gz) was used to compute the lengths in cM. The run time information including the total number of detected IBD segments is included in Additional file 2: Table S3.

The kinship coefficients were computed based on the length of shared IBD segments. The kinship coefficients in chr22 were calculated by summing the lengths of the IBD segments and dividing by four times the length of the chr22 and the number of possible pairs of individuals between every two populations. For chrX, the sum of the IBD segments was divided by the length of chrX and the possible individual pairs. The kinship coefficient was plotted in normalized log scale for nine ethnic groups using 5 and 10 cM results.

For the analysis of distributions of shared IBD segments between different related individuals, the inferred relatedness data from whole genome genotypes generated by KING [20] were downloaded from the UK Biobank project [19]. The relatedness contains related individuals in the UK Biobank up to third-degree relationship. To distinguish parent/offspring and full siblings in the first-degree relationships, IBS0 cutoff of 0.002 was selected. IBS0 in KING denotes the proportion of SNPs with zero identical by state (IBS).

For comparison of the detected pairs with KING results, we considered the pairs sharing an IBD segment more than 40 cM in chromosome 22 and chrX using 5 cM results. The total number of pairs sharing and IBD segment ≥ 40 cM and those that were also reported in the KING relatedness results were extracted and plotted using 10 cM step size. While we did not run the KING program to generate the relatedness call results of UK Biobank, we run KING for the chr 22 data of UK Biobank, and it took about 12 h on a single core. This is not surprising as KING is based on pairwise comparison and scales up quadratically with sample size.

## Additional files

Additional file 1: Supplementary Figures S1–S9. (PDF 1307 kb)

Additional file 2: Supplementary Tables S1–S4. (XLSX 26 kb)

## Data Availability

Source code of RaPID is available at https://github.com/ZhiGroup/RaPID [24] under the GNU General Public License version 2. The source code of the version of RaPID used in this study (version 1.2.3) is archived with DOI: http://doi.org/10.5281/zenodo.3266342 [25] under the GNU General Public License version 2.
