# Supplementary material for: RaPID: ultra-fast, powerful, and accurate detection of segments identical by descent (IBD) in biobank-scale cohorts
Source: Genome Biol. 2019 Jul 25;20:143. doi: 10.1186/s13059-019-1754-8 (PMC6659282; doi:10.1186/s13059-019-1754-8)
Supplement: Supplementary file 1 — Supplementary Figures S1–S9. (PDF 1307 kb) [file 13059_2019_1754_MOESM1_ESM.pdf]

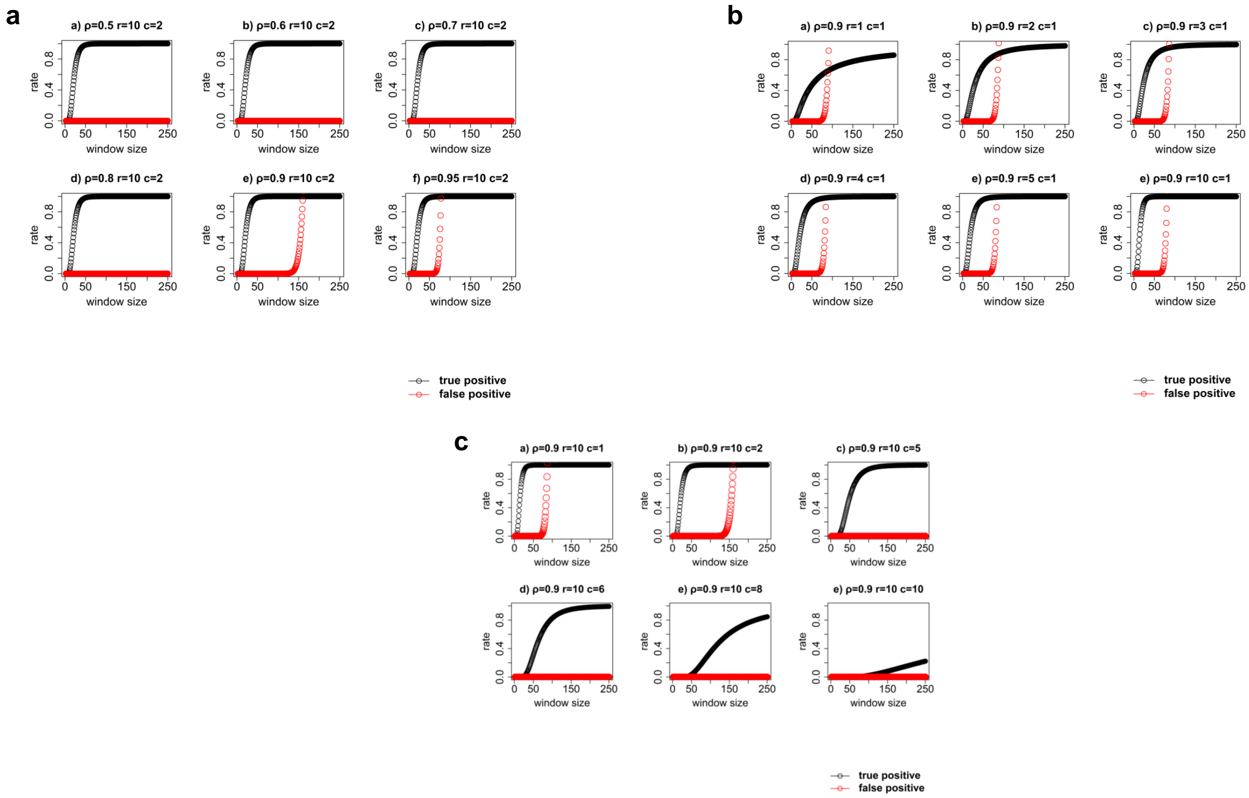

Fig. S1: (**a**) Expected true/false positive rates of RaPID based on the binomial cumulative distribution function for detecting IBD segments with the length 1.5 cM among 4000 haplotypes with genotype error rate of 0.0025 using different probabilities of a random match of haplotypes from 0.5 to 0.95. (**b**) Expected true/false positive rates of RaPID based on the binomial cumulative distribution function for detecting IBD segments with the increasing number of runs among 4000 haplotypes (c = 1, rho = 0.9). (**c**) Expected true/false positive rates of RaPID based on the binomial cumulative distribution function (r = 10, c = 2) with the increasing number of successes for detecting IBD segments with the length 1.5 cM among 4000 haplotypes with the probability of random match of haplotypes of 0.9.

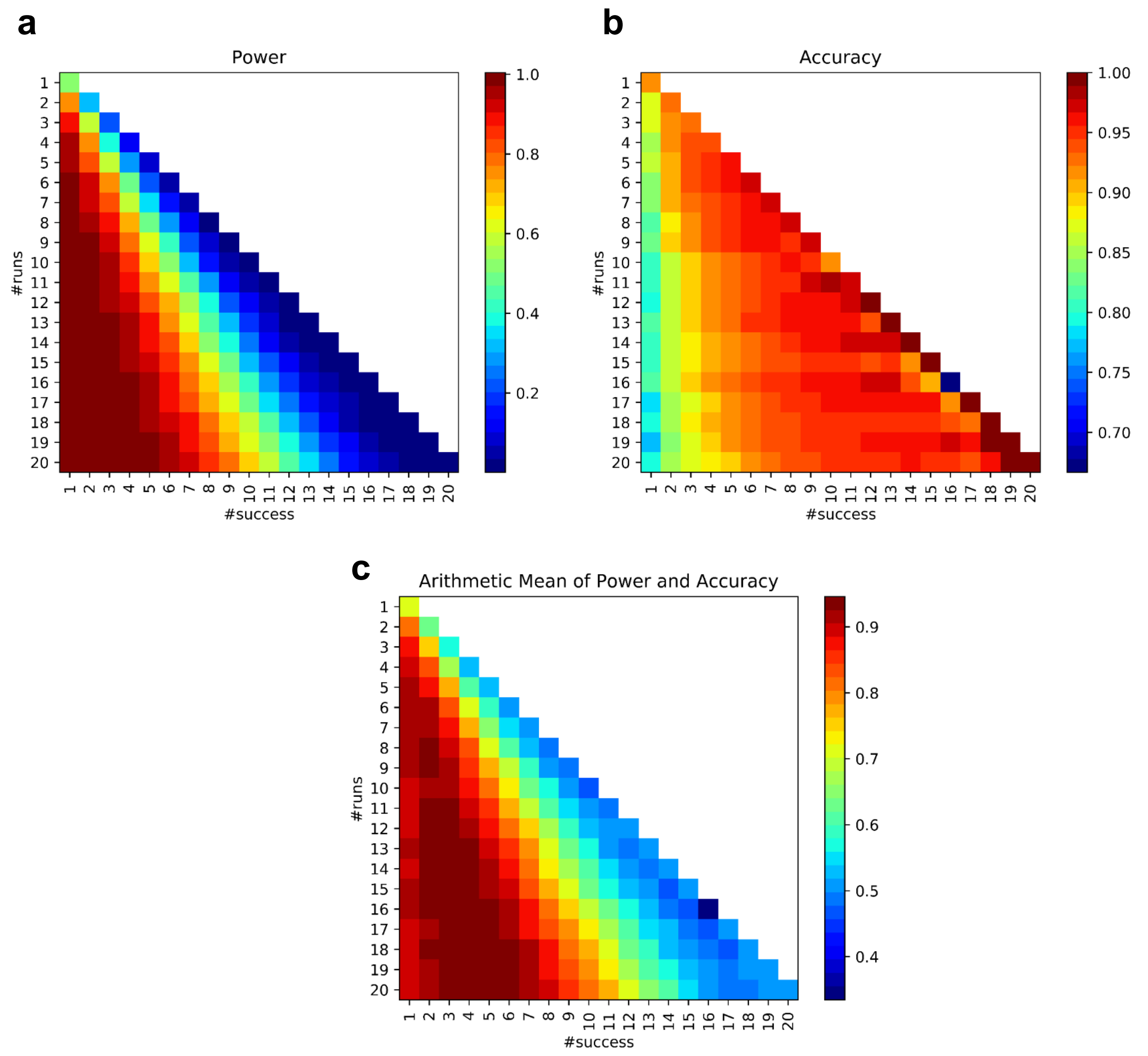

Fig. S2: Effect of using the different number of runs on detection power (Supplementary Figure). (**a**) Detection power values with the different number of PBWT runs (*r*) and success (*c*) using 4k simulated haplotypes. The detection power increases with increasing the number of runs. (**b**) Accuracy values with the different number of PBWT runs (*r*) and success (*c*) using 4k simulated haplotypes. The accuracy increases with increasing the number of successes. (**c**) The arithmetic mean of accuracy and power. A wide range of *r* and *c* values will result in high accuracy and detection power.

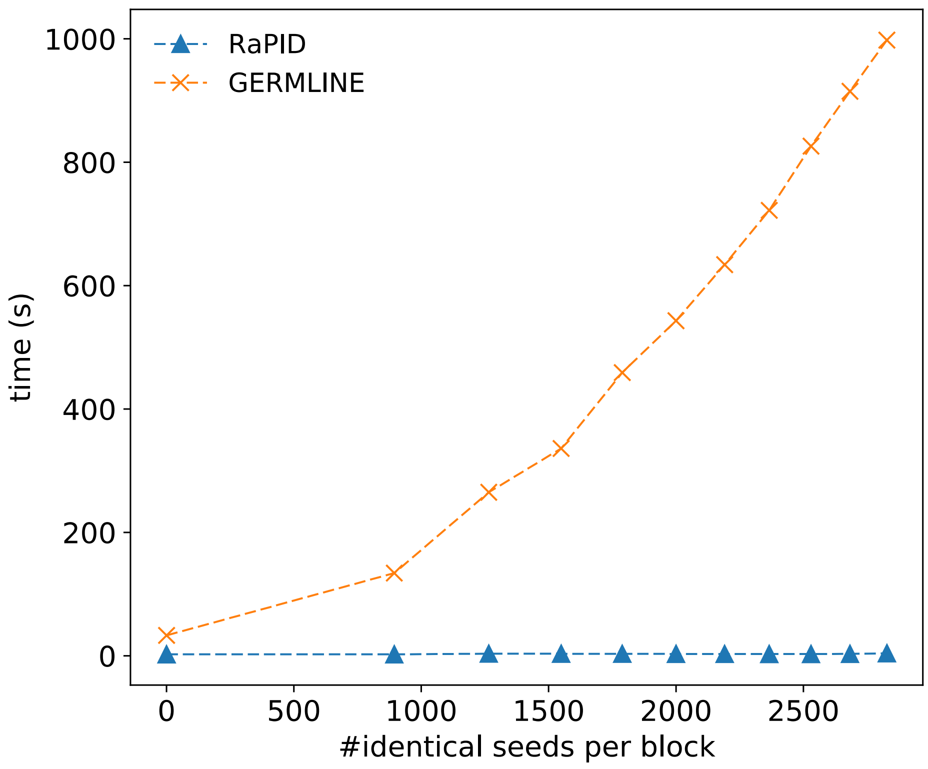

Fig. S3: Comparison of run time of GERMLINE and RaPID for finding IBD segments more than 1.5 cM by implanting exact seed matches in windows of size 128. The number of haplotypes were 4000.

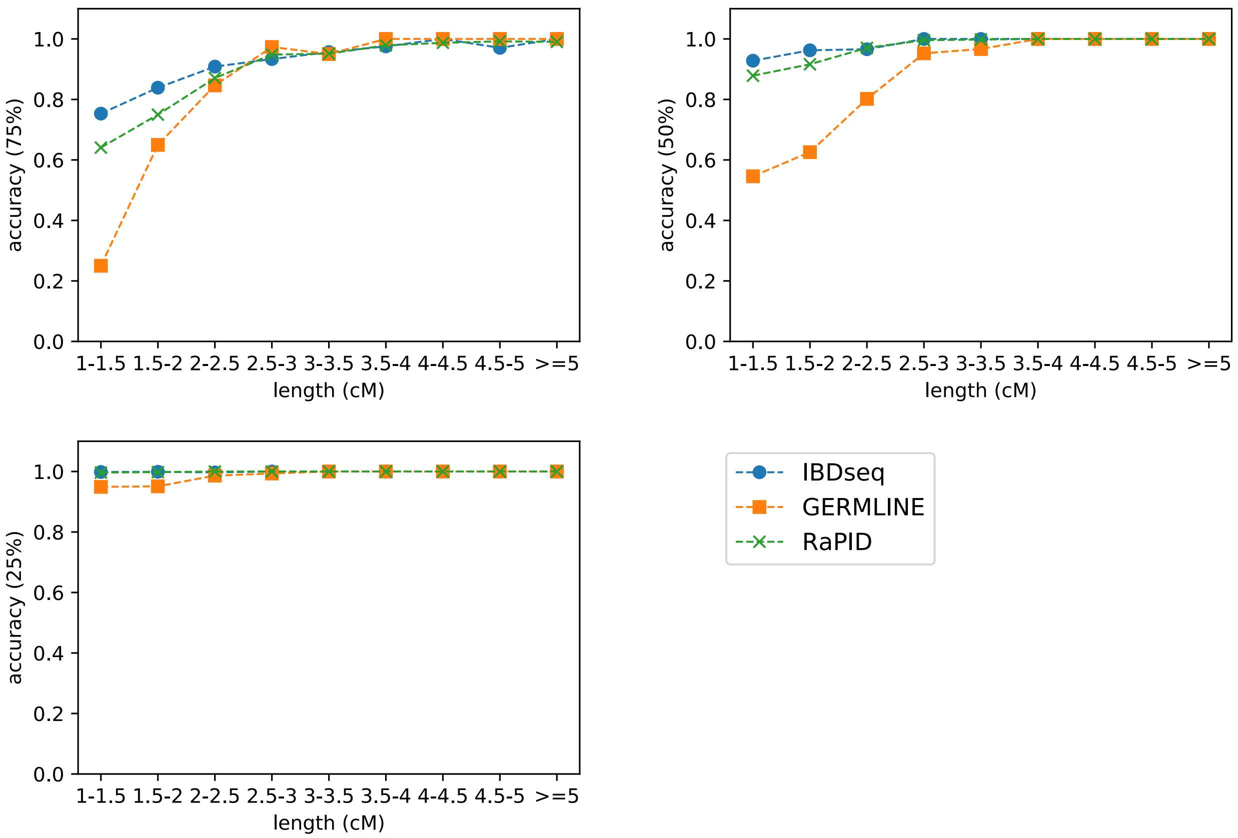

Fig. S4: Accuracy values in the simulated sequencing platform using different cut-offs: 75% (**a**), 50% (**b**) and 25%(**c**). Accuracy is defined as percentage of correctly detected IBD segments. An IBD segment is detected correctly if a proportion of the reported segment overlaps with a true IBD segment and the proportion of covered segment (by only one true IBD) exceeds a given cut-off. As shown in **c**, at least 25% of almost all reported segments by all three tools overlap with a true IBD segment. The accuracy of IBDseq remains higher with larger cut-off values compared to RaPID and GERMLINE.

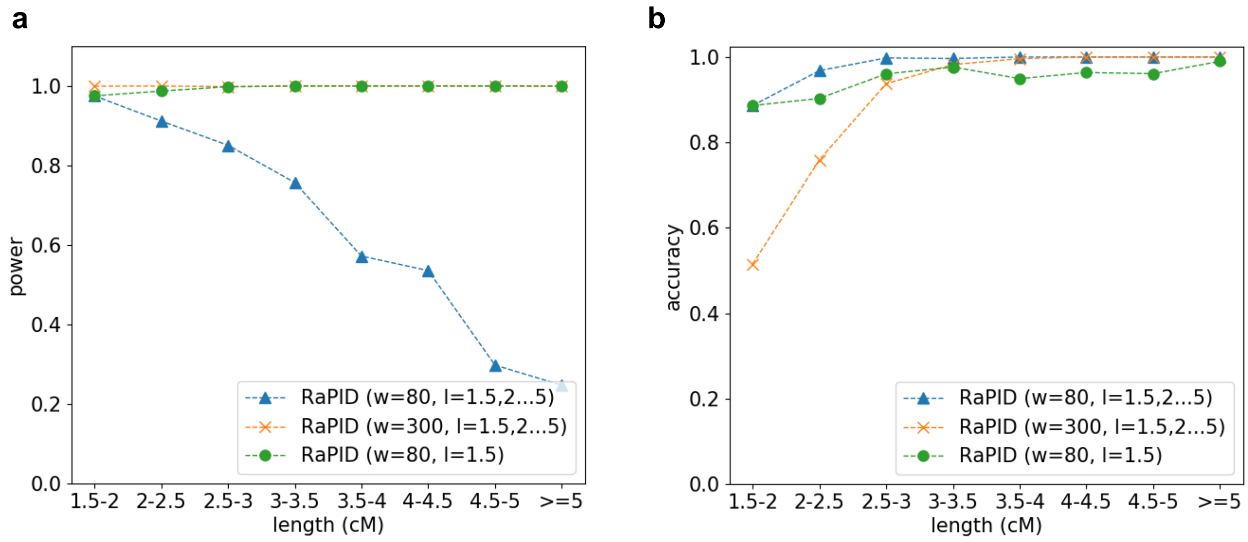

Fig. S5: The effect of using different window sizes and target lengths on detection power (**a**) and accuracy (**b**). In our simulation, RaPID with parameters optimized for 5.0 cM (w = 300) had lower accuracy for detecting 1.5cM segments. RaPID with parameters optimized for 1.5 cM (w = 80) had lower power for detecting 5.0 cM segments if the minimum target length is set to 5 cM. Detection power and also accuracy are high for larger target lengths when the minimum target length is set to 1.5 cM and optimized window size for 1.5 cM (w=80) is used.

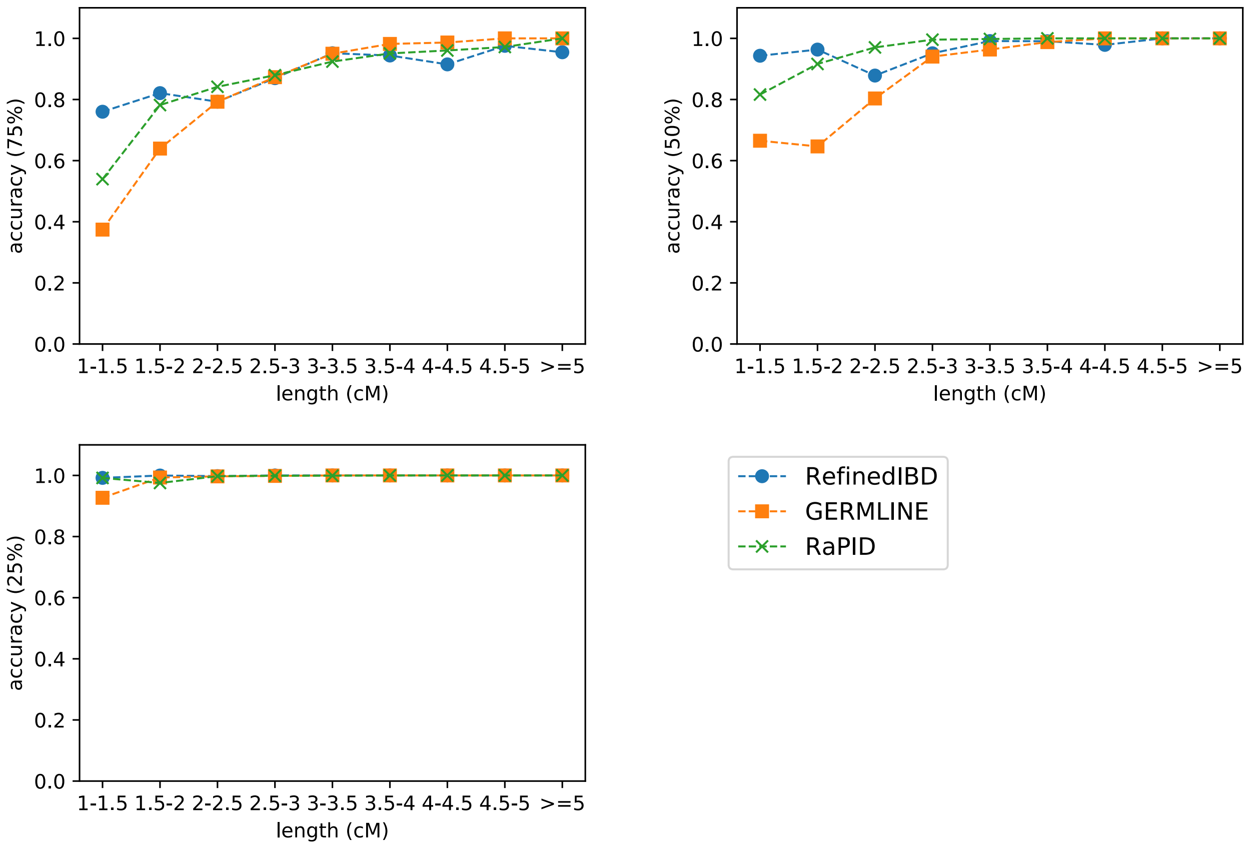

Fig. S6: Accuracy values in the simulated genotyping platform using different cut-offs: 75% (**a**), 50% (**b**) and 25%(**c**). Accuracy is defined as percentage of correctly detected IBD segments. An IBD segment is detected correctly if a proportion of the reported segment overlaps with a true IBD segment and the proportion of covered segment (by only one true IBD) exceeds a given cut-off. As shown in **c**, at least 25% of almost all reported segments by all three tools overlap with a true IBD segment. The accuracy of RefinedIBD remains higher with larger cut-off values compared to RaPID and GERMLINE.

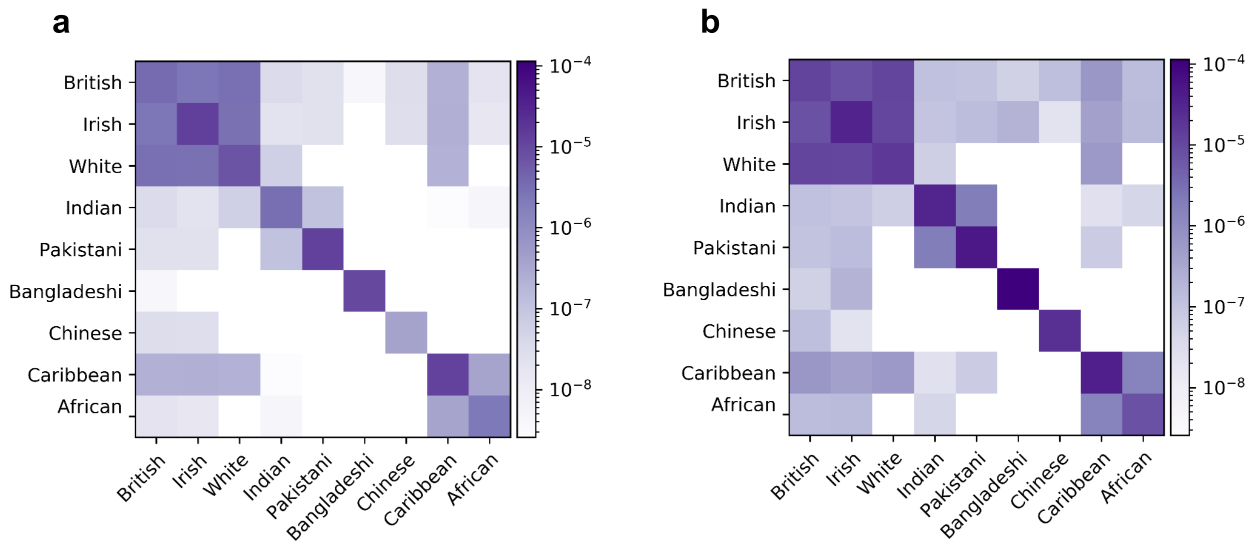

Fig. S7: Kinship values (in log scale) among different ethnic groups in the UK-Biobank using detected IBD segments with the length 10 cM and above in chromosome 22 (**a**), and chromosome X (**b**). The ethnicity groups were extracted using self-reported data by UK-Biobank participants.

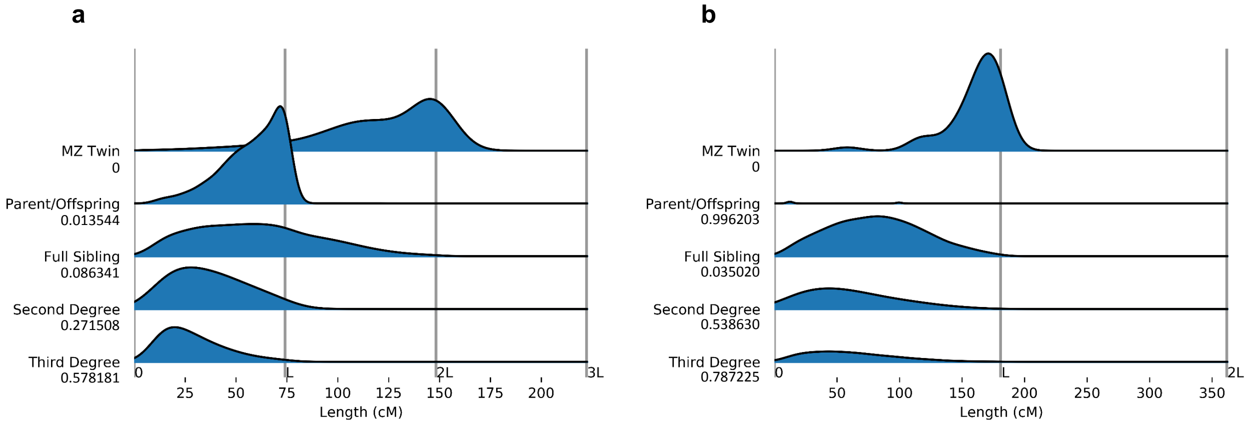

Fig. S8: The probability distributions of the sum of genetic lengths shared among pairs of individuals in 5 degrees of relatedness using detected IBD segments by RaPID with the target length10 cM and above in chromosome 22 (**a**) and chromosome X using male samples (**b**). The numbers below each category show the proportion of pairs for which no IBD segment over 5cM were detected by RaPID. L denotes the total length of the chromosome in cM. Plots were generated by joyplot python package with Gaussian kernels smoothing (default gaussian_kde of scipy). L denotes the total length of the chromosome in cM.

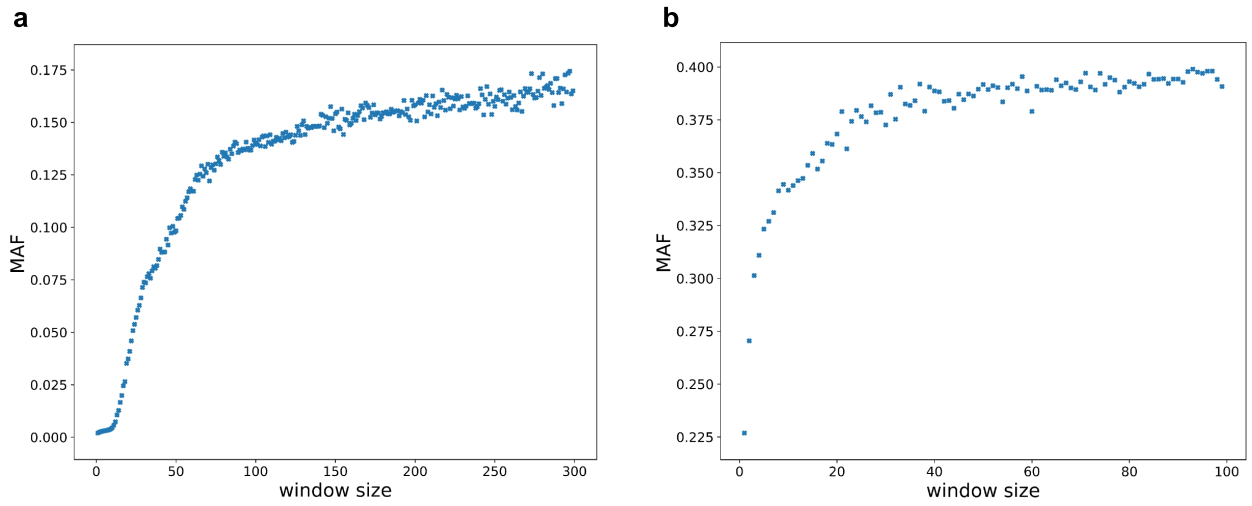

Fig. S9: Expected 10-percentiles of minor allele frequencies (MAFs) values using different window sizes in the simulated sequencing data (**a**) and simulated SNP array data (**b**) containing 4k haplotypes.
